# Associations of Fc gamma receptor (FcgR2a, FcgR3a and FcgR2c) genotype with outcome in metastatic renal cell carcinoma (mRCC) patients receiving high dose interleukin 2 (HD-IL2)

**DOI:** 10.1186/2051-1426-3-S2-P300

**Published:** 2015-11-04

**Authors:** Amy K Erbe, Wei Wang, Kyung Mann Kim, Laskeesha Carmichael, MIkayla Gallendberger, Dustin Hess, Eneida A Mendonca, Yiqiang Song, Jacquelyn A Hank, Su-Chun Chen, Sabina Signoretti, Michael B Atkins, Alexander Carlson, James Mier, David Panka, David F McDermott, Paul M Sondel

**Affiliations:** 1 Department of Human Oncology, University of Wisconsin-Madison, Madison, WI, USA; 2 Department of Biostatistics and Medical Informatics, University of Wisconsin-Madison, Madison, WI, USA; 3 Department of Biostatistics and Medical Informatics, Department of Pediatrics, University of Wisconsin-Madison, Madison, WI, USA; 4 Department of Biostatistics, Dana Farber Cancer Institute, Boston, MA, USA; 5 Department of Pathology, Brigham and Women's Hospital, Boston, MA, USA; 6 The Cytokine Working Group; Department of Medicine, Georgetown-Lombardi Comprehensive Cancer Center, Washington, DC, USA; 7 Division of Hematology/Oncology, Beth Israel Deaconess Medical Center, Boston, MA, USA; 8 The Cytokine Working Group; Division of Hematology/Oncology, Beth Israel Deaconess Medical Center, Boston, MA, USA; 9 Department of Human Oncology, Department of Pediatrics, University of Wisconsin-Madison, Madison, WI, USA

## 

HD-IL2 was given to patients with mRCC in a prospective trial (ClinicalTrials.gov ID: NCT00554515) that demonstrated an overall response rate of 25%. To identify predictors of response, we genotyped patients for single nucleotide polymorphisms (SNPs) in FcgR genes: FcgR2a, FcgR3a, and FcgR2c. These FcgRs are variably expressed on immune cells, and bind to the IgG portion of antibodies, triggering activation.

FcgR2a [SNP = histidine/arginine (H/R); expressed on neutrophils, monocytes-macrophages and antigen-presenting cells (APCs)] and FcgR3a [SNP = valine/phenylalanine (V/F); expressed on NK cells and some APCs] impact patient response to immunotherapy [mainly monoclonal antibody-based (mAb) therapies] in several malignancies, as both FcgR2a-H and FcgR3a-V have higher antibody binding affinity than FcgR2a-R or FcgR3a-F, respectively. FcgR2c, also expressed on NK cells, has a SNP that regulates its expression [C nucleotide = expression; T nucleotide = non-expression (C/T)]. About 20-40% of individuals express FcgR2c; little is known about the role of FcgR2c expression in cytokine-based immunotherapy.

We found associations of FcgR genotypes with patient response to HD-IL2. Dual combination analyses of FcgR3a with FcgR2a genotypes revealed significantly improved %Tumor Shrinkage in patients with either FcgR3a-VV and/or FcgR2a-HH (n=34) as compared to those patients genotyped as FcgR3a-VF or FF with FcgR2a-HR or RR (n=70) (p=0.047). For analyses including both FcgR3a and FcgR2c genotypes, significantly improved OS was seen in those with ≥2 alleles of either FcgR3a-V and/or FcgR2c-C (n=27) vs. those with < 2 alleles of either FcgR3a-V and/or FcgR2c-C (n=79) (p=0.013). We further considered combinations that included the genotypes of all 3 genes; we identified 42 patients with “favorable” genotypes and 64 with “unfavorable”. We saw significant improvement in the %Tumor shrinkage (p = 0.033) and a trend for improvement in OS (p = 0.071) in the “favorable genotype” group.

As reported previously by others, some cancer patients make endogenous anti-tumor Ab. The association of these “favorable” FcgR genotypes with outcome suggests there may be a beneficial interaction of “favorable” FcR genotypes with endogenous anti-tumor Ab. Favorable FcgRs may support the induction of *in vivo* antibody-dependent cell-mediated cytotoxicity (ADCC) or antibody-dependent cellular phagocytosis (ADCP). In addition, ADCP by APC with a favorable pattern of FcgRs, may be playing a role in enhancing induction of effective adaptive immunity, via augmented antigen presentation. Further analyses are needed to validate these exploratory findings and clarify the mechanisms by which these favorable FcgR genotypes are associated with improved outcome in these patients with mRCC treated with HD-IL2.

